# Serum vitamins and inflammatory bowel disease in children: a case-control study

**DOI:** 10.3389/fimmu.2026.1903865

**Published:** 2026-10-26

**Authors:** Xiaojuan Li, Tiewei Li, Yuesheng Wang, Yuanzhe Li, Shuai Zhang, Wenbo Yan, Yushu Ma, Ziying Li, Junmei Yang, Zhipeng Jin

**Affiliations:** 1 Department of Clinical Laboratory, Zhengzhou Key Laboratory of Children’s Infection and Immunity, Children’s Hospital Affiliated to Zhengzhou University, Henan Children’s Hospital, Zhengzhou Children’s Hospital, Zhengzhou, China; 2 Department of Gastroenterology, Children’s Hospital Affiliated to Zhengzhou University, Henan Children’s Hospital, Zhengzhou Children’s Hospital, Zhengzhou, China; 3 Department of Children’s Health Management Center, Children’s Hospital Affiliated to Zhengzhou University, Henan Children’s Hospital, Zhengzhou Children’s Hospital, Zhengzhou, China; 4 Department of Paediatric Intensive Care Unit, Children’s Hospital Affiliated to Zhengzhou University, Henan Children’s Hospital, Zhengzhou Children’s Hospital, Zhengzhou, China

**Keywords:** children, Crohn’s disease, inflammatory bowel disease, ulcerative colitis, vitamin

## Abstract

**Background:**

Micronutrient deficiencies are common in pediatric inflammatory bowel disease (IBD), yet data on multiple vitamin profiles remain limited. This study aimed to comprehensively evaluate serum vitamin levels in children with IBD and explore their associations with immune cell profiles.

**Methods:**

A retrospective observational study was conducted at the Children’s Hospital Affiliated to Zhengzhou University. A total of 46 children diagnosed with IBD who had vitamin profiling were enrolled between March 2022 and March 2026. A control group of 46 healthy children, who underwent health check-ups and vitamin assessments during the same period, were also included, matched 1:1 for age and sex. Clinical and laboratory data were retrieved from the hospital’s electronic medical record system. Serum levels of vitamins A, D, E, K, B1, B2, B3, B5, 5-methyltetrahydrofolate, and C were measured using ultra-high performance liquid chromatography–tandem mass spectrometry.

**Results:**

Compared with controls, IBD patients had significantly lower vitamin A (VA) and vitamin C (VC) levels, while vitamin E (VE) levels were higher (all P < 0.05). Deficiency rates of VA (76.1% vs. 28.3%), VC (50.0% vs. 26.1%), and vitamin D (41.3% vs. 21.7%) were significantly increased in the IBD group (all P < 0.05). Within the IBD cohort, VA, VD, and VC levels correlated significantly with neutrophil and lymphocyte percentages. Multivariate logistic regression identified lower VA level (OR = 0.159, 95% CI: 0.032-0.792, P = 0.025) and VA deficiency (OR = 7.800, 95% CI: 1.735–35.055, P = 0.007) as factors independently associated with IBD.

**Conclusions:**

Pediatric IBD patients exhibit significant deficiencies in VA, VC, and VD, and within the IBD cohort, these vitamins (VA, VD, and VC) correlate significantly with neutrophil and lymphocyte percentages. Multivariate regression suggests a potential independent association between VA status and pediatric IBD, though these are preliminary observational findings. This study provides preliminary multi-vitamin profiling data for pediatric IBD, highlighting the need for attention to VA and VC deficiencies alongside routine VD monitoring. Further validation in larger multicenter cohorts is required.

## Introduction

Inflammatory bowel disease (IBD), encompassing ulcerative colitis (UC) and Crohn’s disease (CD), is characterized by chronic, relapsing intestinal inflammation with alternating active and remitting phases (1). Although the precise etiology remains incompletely understood, current evidence supports a complex interplay of environmental factors, genetic predisposition, aberrant immune responses, and gut microbiota dysbiosis in disease initiation and progression (2). Globally, the incidence of pediatric-onset IBD has been steadily increasing in most countries, with emerging cases now reported in previously unaffected regions (3, 4). However, epidemiological data on childhood IBD from developing countries remain scarce. Concurrently, shifts in environmental exposures, increased public awareness of the disease, and improved access to specialized diagnostic services and endoscopy have contributed to a decreasing age at diagnosis for pediatric IBD (5–7).

Malnutrition is a highly prevalent clinical issue in children with IBD, with micronutrient deficiencies affecting over 50% of these patients (8). Accumulating clinical evidence indicates that poor nutritional status and micronutrient deficiencies not only impair childhood and adolescent growth and development but are also closely associated with reduced treatment response, increased risk of disease relapse, and adverse outcomes (7, 9, 10). Recent years have seen a shift from single-vitamin focus to broader concerns: a 2024 study showed that vitamin D receptor (*VDR*) polymorphism had significantly increased IBD risk, and *flavobacterium okeanokoites I* (*FokI*) polymorphism was associated with disease activity in UC children (11). A 2025 study in newly diagnosed pediatric IBD patients revealed that 85% of UC and 81% of CD patients had sarcopenia, with severe vitamin D (VD) deficiency present in 35.9%-38.5%; children with more severe sarcopenia had significantly lower VD levels (12). Childhood and adolescence represent critical windows for growth and development. Maintaining adequate vitamin levels is essential for proper physical growth, skeletal development, immune system maturation, and neurocognitive function (13–15). Emerging mechanistic insights indicate that vitamin A (VA) maintains T helper 1 cell (Th1)/T helper 2 cell (Th2) balance via retinoic acid (RA)-mediated *forkhead box P3* (*FOXP3*)+ Treg regulation (16), while vitamin C (VC) deficiency may exacerbate oxidative stress-driven mucosal injury (17), yet studies linking these vitamin levels to circulating immune cell profiles in pediatric IBD are lacking.

Current national and international guidelines and consensus statements recommend vitamin status assessment as part of nutritional evaluation in pediatric patients with IBD, with particular emphasis on vitamin D (18–20). Despite these recommendations, existing evidence on micronutrient deficiencies in pediatric IBD is unevenly distributed. Most vitamin-related studies in this population have focused on VD, vitamin B12, iron, and zinc, leaving substantial gaps in knowledge regarding other vitamins—particularly as relevant investigations have remained sparse over recent decades (21, 22). Of note, evidence on VC deficiency largely derives from adult IBD cohorts (17), and protective effects of vitamin E (VE) are mainly described in animal models (23). Moreover, the application of ultra-high performance liquid chromatography-tandem mass spectrometry (UHPLC-MS/MS) for simultaneous quantification of 10 fat- and water-soluble vitamins has not been systematically used in pediatric IBD. Moreover, studies examining the relationships between vitamin levels and circulating immune cell profiles in children with IBD are notably lacking. Therefore, addressing these unmet needs, the present study aims to comprehensively evaluate multiple vitamin levels in pediatric patients with IBD, using a validated ultra-high performance liquid chromatography–tandem mass spectrometry (UHPLC–MS/MS) panel, thereby providing a foundation for optimizing clinical nutritional assessment and establishing a disease-specific vitamin screening protocol for this vulnerable population, while also elucidating potential associations between various vitamins and disease characteristics. Specifically, this is the first study to simultaneously analyze 10 serum vitamins under a strict age- and sex-matched case-control design and to systematically explore their correlations with neutrophil and lymphocyte percentages, aiming to fill the current gap in multi-vitamin profiling for pediatric IBD.

## Methods

### Study population

This retrospective case-control study was conducted at the Children’s Hospital Affiliated to Zhengzhou University. Among 56 eligible pediatric patients with IBD during the study period, 46 underwent multiplex vitamin profiling and were consecutively enrolled, including 25 with CD and 21 with UC; the remaining 10 underwent only solitary vitamin D testing and were excluded because other vitamins were not measured. In routine clinical practice, vitamin profiling is recommended after IBD diagnosis according to current national and international guidelines/consensus (18–20). This recommendation was communicated to all pediatric patients with IBD, without selective communication or preferential recommendation for only some patients; testing was performed only with guardian consent and was not mandatory. The multiplex panel allows multiple vitamins to be measured simultaneously. Fat-soluble vitamins (A, D, E, K) and water-soluble vitamins (B1, B2, B3, B5, 5-methyltetrahydrofolate [5-MTHF], and C) were quantified using UHPLC–MS/MS (24). Inclusion criteria for IBD cases were: (1) confirmed diagnosis of IBD; (2) age < 18 years; and (3) availability of complete clinical data in the electronic medical record system, including disease type, endoscopy findings, and laboratory results. Exclusion criteria were: (1) prior surgical treatment for IBD; (2) incomplete clinical data; (3) immunodeficiency or other immune-related disorders; (4) prior vitamin supplementation therapy.

To ensure population homogeneity and minimize confounding, healthy control children were recruited from the Pediatric Health Management Clinic at our hospital during the same period (March 2023 to March 2026). Given that age, sex, and season influence vitamin levels, controls were matched 1:1 to cases based on these key parameters, yielding 46 control children. Children who had received vitamin supplementation prior to their health examination were excluded. The study protocol was approved by the Ethics Committee of Henan Children’s Hospital (approval No. 2024-079-002) and complied with the Declaration of Helsinki. All procedures, including vitamin level measurements performed as part of routine clinical care at the request of patients or their guardians, constituted standard clinical practice. Data were collected retrospectively, and strict anonymization protocols ensured privacy protection. Owing to the retrospective nature of the study, the ethics committee waived the requirement for informed consent (approval No. 2024-079-002).

### Diagnosis of IBD

All children with IBD were diagnosed according to the “Consensus Opinion on Diagnostic Criteria for Pediatric Inflammatory Bowel Disease” issued by Chinese Medical Pediatric Association, Gastroenterology Group (25). Patients were classified into UC and CD based on clinical, endoscopic, radiological, and histopathological findings, after excluding intestinal tuberculosis and other chronic intestinal disorders. For UC, diagnosis required persistent (>4 weeks) or recurrent bloody or mucopurulent diarrhea, abdominal pain, tenesmus, weight loss, and extraintestinal manifestations. Colonoscopy showed continuous, diffuse inflammation starting from the rectum, with loss of vascular pattern, friability, erosions, or ulcers. Histopathology confirmed cryptitis, crypt abscesses, goblet cell depletion, and architectural distortion. For CD, key features included recurrent abdominal pain, diarrhea, growth retardation, perianal lesions, and fistulas. Endoscopy revealed discontinuous, segmental, skip lesions with aphthous or serpiginous ulcers and a cobblestone appearance. Radiology identified strictures, fistulas, and segmental bowel wall thickening. Histopathology verified transmural inflammation and non-caseating granulomas. Definite diagnosis was established using the World Health Organization (WHO)-recommended criteria (non-caseating granuloma plus two other features). All diagnoses were independently confirmed by two pediatric clinical gastroenterologists.

### Data collection

Clinical, anthropometric, laboratory, and disease-specific data were retrospectively extracted from the electronic medical records of all participants. The following variables were collected: age, sex, area of residence (urban vs. rural), body mass index (BMI), systolic blood pressure (SBP), diastolic blood pressure (DBP), white blood cell count (WBC), neutrophil percentage, lymphocyte percentage and monocyte percentage. Serum concentrations of multiple vitamins were also extracted, including VA, VD, VE, vitamin K1 (VK1), vitamin B1 (VB1), vitamin B2 (VB2), vitamin B3 (VB3), vitamin B5 (VB5), 5-MTHF, and VC. All peripheral blood routine indicators were measured using a Sysmex NX-2800 automated CBC analyzer (Sysmex Corporation, Japan), following the manufacturer’s standard protocols. The laboratory strictly implemented internal quality control procedures and passed the national external quality assessment in China, ensuring stable and reliable hematological test results. Vitamin levels were quantified by UHPLC-MS/MS, and the detailed analytical procedures have been described elsewhere (24).

### Definitions of vitamin deficiency

Deficiency cutoffs were defined according to established references. In accordance with the “Expert Consensus on Clinical Application of Vitamin A and Vitamin D for Children in China (2024)” (26), VA deficiency is defined as levels below 1.05μmol/L, and VD deficiency as levels below 30 nmol/L. Because no uniform international or domestic guidelines exist defining deficiency thresholds for VE, VC, and B vitamins. Therefore, the thresholds for insufficiency used in this study were established based on reference ranges provided by leading clinical laboratories, including Mayo Clinic, Labcorp, and Quest Diagnostics. Specific cutoff values were defined as follows: VE deficiency: < 3.8 mg/mL (Mayo Clinic); VB1 deficiency: < 2.12 ng/mL (Quest Diagnostics); VB2 deficiency: < 1 ng/mL (Mayo Clinic); VB3 deficiency: < 5.2 ng/mL (Labcorp); VB5 deficiency: < 3.45 ng/mL for ages 0–10 years and < 37 ng/mL for ages > 10 years (Mayo Clinic); VB7 deficiency: < 0.1 ng/mL for ages < 12 years and < 0.22 ng/mL for ages ≥ 12 years (Mayo Clinic); 5-MTHF deficiency: < 4 ng/mL (Mayo Clinic); VC deficiency: < 4 mg/mL (Mayo Clinic).

### Statistical analysis

The data analysis for this study began in May 2026. All statistical analyses were carried out using SPSS version 24.0 (IBM Corp., Armonk, NY, USA). The normality of continuous variables was examined by the Shapiro-Wilk test. Data following a normal distribution are presented as mean ± standard deviation (SD) and were compared between groups using the independent-samples t-test. Non-normally distributed data are expressed as median with interquartile range (IQR) and were analyzed with the Mann-Whitney U test. Categorical variables (e.g., sex, vitamin deficiency status) are reported as frequencies and percentages, and group comparisons were performed using the chi-square test or Fisher’s exact test, as appropriate. Spearman’s rank correlation coefficient was used to evaluate associations between vitamin levels and inflammatory immune cell parameters. Univariate logistic regression was first performed to screen for factors potentially associated with the outcome; variables with a significant association (P < 0.05) in the univariate analysis were then entered into a multivariable logistic regression model to identify independent predictors. *Post hoc* power analysis was performed for primary endpoints (serum vitamin concentrations and vitamin deficiency status) with two-sided α=0.05 and power ≥0.80 defined as adequate; power for non-normal continuous variables was approximated by Cohen’s d under normality assumption as reference values, and categorical variables were calculated using Cohen’s h and non-central chi-square distribution. Post-hoc power mainly aids interpretation of non-significant findings, and low power for significant results indicates potential effect-size overestimation rather than invalidating P-values. All tests were two-sided, and a P-value < 0.05 was considered statistically significant.

## Result

### Study population characteristics

A total of 92 subjects were enrolled in this study, including 46 pediatric patients with IBD (25 males [54.3%] and 21 females [45.7%]; median age: 12.25 years [interquartile range: 9.40–13.50]; age range: 5.33–16.00 years). Among the IBD group, 25 patients were diagnosed with CD and 21 with UC. Baseline characteristics of each group are summarized in **Table 1**. No statistically significant differences were observed between the IBD group and the control group in terms of age, sex distribution, body mass index (BMI), diastolic blood pressure, white blood cell count, or neutrophil percentage (all P > 0.05). Regarding geographic distribution, the control group predominantly consisted of urban residents (84.8%), whereas a significantly higher proportion of rural residents was found in the IBD group (52.2%), with a statistically significant difference (P < 0.001). Furthermore, systolic blood pressure, heart rate, lymphocyte percentage, and monocyte percentage differed significantly between the IBD group and the control group (all P < 0.005). Comparisons between the CD and UC revealed no significant differences in age, sex, geographic distribution, BMI, blood pressure levels, and any immune cell parameters (all P > 0.05). Of note, post-hoc power analysis indicated that most CD-versus-UC comparisons exhibited insufficient statistical power (<0.80); therefore, these negative findings should be interpreted with caution.

**Table 1:** Basic characteristics of study subjects.

Variables	Control (n = 46)	IBD (n = 46)	P	*Post-hoc* power	IBD			*Post-hoc* power
					CD(n = 25)	UC (n = 21)	^#^P	
Age (years)	12.0 (9.0, 13)	12.25 (9.40, 13.50)	0.226	0.164	12.25 (8.34, 13.75)	12.25 (11.04, 13.25)	0.551	0.224
Male, n (%)	25 (54.3%)	25 (54.3%)	1	0.050	12 (57.1%)	13 (52.0%)	0.727	0.064
Residential classification			<0.001	0.974			0.979	0.050
Urban, n (%)	39 (84.8%)	22 (47.8%)			12 (48.0%)	10 (47.6%)		
Rural, n (%)	7 (15.2%)	24 (52.2%)			13 (52.0%)	11 (52.4%)		
BMI	17.05 (15.62, 20.56)	16.57 (14.44, 19.02)	0.205	0.314	16.72 (15.01, 18.97)	15.91 (13.74, 19.15)	0.396	0.096
SBP (mmHg)	104 (98, 110)	96 (93, 105)	0.001	0.827	95 (92, 103)	98 90 (93, 105)	0.348	0.159
DBP (mmHg)	68.0 (64, 73)	66 (61, 69)	0.112	0.400	66 (59, 69)	66 (62, 71)	0.634	0.110
Heart rate (rate/min)	84.0 (76.0, 92.5)	93.5 (86.8, 100.3)	<0.001	0.952	97.0 (90.5, 101.5)	92.0 (82.0, 97.0)	0.139	0.151
WBC	6.48( 5.70, 7.46)	7.01 (5.62, 8.70)	0.052	0.651	7.04 (6.28, 9.72)	6.98 (5.19, 8.21)	0.316	0.084
Neutrophil (%)	53.00 (49.15, 60.45)	58.40 (49.38, 67.63)	0.184	0.277	57.70 (50.35, 68.20)	59.10 (42.45, 65.45)	0.408	0.160
Lymphocyte(%)	41.15 (33.40, 44.00)	33.00 (26.28, 41.08)	0.012	0.706	29.40 (24.40, 40.35)	33.80 (26.35, 48.20)	0.337	0.211
Monocyte (%)	2.85 (2.50, 3.33)	5.05 (4.05, 7.60)	<0.001	1.000	5.40 (3.95, 7.95)	4.90 (4.10, 6.25)	0.596	0.224

IBD, inflammatory bowel disease; UC, ulcerative colitis; CD, Crohn's disease; BMI, body mass index; SBP, systolic blood pressure; DBP, diastolic blood pressure; WBC, white blood cell count.

^#^ P < 0.05 for the comparison of disease activity between the CD and UC groups.

### Vitamin levels and nutritional status in two groups of children

Analysis of peripheral blood vitamin levels in the two groups is presented in **Table 2**. Compared with the control group, pediatric patients with IBD exhibited significantly lower concentrations of VA and VC, whereas VE levels were significantly elevated. Although not statistically significant, decreasing trends were observed for VD, VK1, VB1, and 5-MTHF, whereas increasing trends were noted for VB2, VB3, and VB5.Vitamin deficiency rates were further compared between the control and IBD groups, as summarized in **Table 3**. The IBD group showed significantly higher deficiency rates of VA (76.1% vs. 28.3%, P < 0.001), VC (50.0% vs. 26.1%, P = 0.018), and VD (41.3% vs. 21.7%, P = 0.043) compared with the control group. No significant differences were observed in deficiency rates of other vitamins between the two groups. When comparing CD and UC, no significant differences were found in either vitamin levels or deficiency rates. Given the relatively low incidence of pediatric CD and UC and the absence of inter-group differences across clinical, laboratory and vitamin indices within our cohort, CD and UC patients were pooled into the overall IBD cohort for primary analyses. For several variables showing statistical significance between IBD and controls, *post-hoc* power was below 0.80. Low *post-hoc* power does not invalidate statistical P-values but suggests that the observed effect sizes may be overestimated in the present cohort and require validation in larger samples.

**Table 2:** Comparison of multiple vitamin levels between the different groups.

Variables	Control (n = 46)	IBD (n = 46)	P	*Post-hoc* power	IBD			*Post-hoc* power
					CD (n = 25)	UC (n = 21)	P	
VA (μmol/L)	1.31 (1.03, 1.61)	0.87 (0.74, 1.05)	<0.001	0.970	0.83 (0.62, 1.03)	0.93 (0.79, 1.17)	0.229	0.126
VD (nmol/L)	37.73 (31.16, 20.96)	32.51 (23.69, 56.83)	0.251	0.055	33.41 (23.89, 60.78)	32.27 (23.56, 54.35)	0.501	0.111
VE (ug/ml)	5.51 (4.65, 6.70)	6.50 (4.98, 8.14)	0.019	0.595	6.51 (5.15, 8.04)	6.49 (4.35,8.31)	0.938	0.050
VK (ng/ml)	0.42 (0.25, 0.72)	0.32 (0.14, 0.85)	0.587	0.181	0.28 (0.12, 0.76)	0.37 (0.16,0.98)	0.559	0.075
VB1 (ng/ml)	2.89 (2.03, 3.84)	2.78 (1.67, 3.95)	0.719	0.060	2.95 (2.03, 4.10)	2.70 (1.39, 3.20)	0.265	0.307
VB2 (ng/ml)	7.42 (6.17, 11.00)	8.56 (5.82, 13.42)	0.548	0.133	8.69 (6.39, 14.37)	7.87 (5.03, 11.77)	0.221	0.242
VB3 (ng/ml)	30.63 (16.21, 48.02)	37.19(20.84, 56.96)	0.128	0.395	44.09 (15.56, 66.96)	34.01 (21.44, 45.70)	0.589	0.078
VB5 (ng/ml)	42.46 (34.26, 60.98)	46.83 (29.94, 72.44)	0.988	0.339	49.94 (33.37, 66.96)	45.93 (26.92, 68.70)	0.474	0.117
5-MTHF (ng/ml)	7.64 (3.92, 11.03)	5.41 (2.87, 15.89)	0.601	0.388	4.13 (2.30, 12.57)	8.05 (3.55, 19.46)	0.182	0.068
VC (ug/ml)	7.52 (3.88, 11.26)	4.00 (2.06, 8.67)	0.009	0.375	4.02 (2.03, 7.70)	3.64 (2.07, 9.32)	0.783	0.058

IBD, inflammatory bowel disease; UC, ulcerative colitis; CD, Crohn's disease; VA, vitamin A; VD, vitamin D; VE, vitamin E; VK, vitamin K; VB1, vitamin B1; VB2, vitamin B2; VB3, vitamin B3; VB5, vitamin B5; 5-MTHF, 5-methyltetrahydrofolate; VC, vitamin C.

**Table 3:** Comparison of multiple vitamin status between the different groups.

Variables	Control (n = 46)	IBD (n = 46)	P	*Post-hoc* power	IBD			*Post-hoc* power
					CD(n = 25)	UC (n = 21)	P	
VA status			<0.001	0.998			0.497	0.104
VA sufficiency, n (%)	33 (71.7%)	11 (23.9%)			5 (20.0%)	6 (28.6%)		
VA deficiency, n (%)	13 (28.3%)	35 (76.1%)			20 (80.0%)	15 (71.4%)		
VD status			0.043	0.533			0.592	0.125
VD sufficiency, n (%)	36 (78.3%)	27 (58.7%)			9 (36.0%)	6 (28.6%)		
VD deficiency, n (%)	10 (21.7%)	19 (41.3%)			16(64.0%)	15 (71.4%)		
VE status			0.369	0.147			0.293	0.184
VE sufficiency, n (%)	41 (89.1%)	38 (82.6%)			22 (88.0%)	16 (76.2%)		
VE deficiency, n (%)	5 (10.9%)	8 (17.4%)			3 (12.0%)	5 (23.8%)		
VB1 status			0.815	0.056			0.484	0.108
VB1 sufficiency, n (%)	34 (73.9%)	33 (71.7%)			19 (76.0%)	14 (66.7%)		
VB1 deficiency, n (%)	12 (26.1%)	13 (28.3%)			6 (24.0%)	7 (33.3%)		
VB2 status			1	—			1	—
VB2 sufficiency, n (%)	46 (100%)	46 (100%)			25 (100%)	21 (100%)		
VB2 deficiency, n (%)	0 (0)	0 (0)			0 (0)	0 (0)		
VB3 status			1	—			1	—
VB3 sufficiency, n (%)	46 (100%)	46 (100%)			25 (100%)	21 (100%)		
VB3 deficiency, n (%)	0 (0)	0 (0)			0 (0)	0 (0)		
VB5 status			0.252	0.210			0.292	0.184
VB5 sufficiency, n (%)	35 (76.1%)	30 (65.2%)			18 (72.0%)	12 (57.1%)		
VB5 deficiency, n (%)	11 (23.9%)	16 (34.8%)			7 (28.0%)	9 (42.9%)		
5-MTHF status			0.174	0.277			0.280	0.193
5-MTHF sufficiency, n (%)	35 (76.1%)	29 (63.0%)			14 (56.0%)	15 (71.4%)		
5-MTHF deficiency, n (%)	11 (23.9%)	17 (37.0%)			11 (44.0%)	6 (28.6%)		
VC status			0.018	0.667			0.767	0.060
VC sufficiency, n (%)	34 (73.9%)	23 (50.0%)			13 (52.0%)	10 (47.6%)		
VC deficiency, n (%)	12 (26.1%)	23 (50.0%)			12 (48.0%)	11 (52.4%)		

IBD, inflammatory bowel disease; UC, ulcerative colitis; CD, Crohn's disease; VA, vitamin A; VD, vitamin D; VE, vitamin E; VK, vitamin K; VB1, vitamin B1; VB2, vitamin B2; VB3, vitamin B3; VB5, vitamin B5; 5-MTHF, 5-methyltetrahydrofolate; VC, vitamin C.

### Correlation analysis between vitamins and peripheral blood immune cell in pediatric patients with IBD

Based on the vitamins that showed significant differences in serum levels or deficiency rates between the IBD and control groups (VA, VD, VC, and VE), further correlation analyses were performed with peripheral blood immune cell parameters in pediatric patients with IBD. The results are presented in **Table 4**. VA levels showed a significant negative correlation WBC (r = -0.358, P = 0.015) and neutrophil percentage (r = -0.500, P < 0.001), and a significant positive correlation with lymphocyte percentage (r = 0.502, P < 0.001). VD levels showed a significant negative correlation with neutrophil percentage (r = -0.374, P = 0.011) and a significant positive correlation with lymphocyte percentage (r = 0.396, P = 0.007). No significant correlation was observed between VD levels and WBC count and monocyte percentage (all P > 0.05). VC levels exhibited significant negative correlations with WBC count (r = -0.474, P < 0.001) and neutrophil percentage (r = -0.549, P < 0.001), and a significant positive correlation with lymphocyte percentage (r = 0.512, P < 0.001). No significant correlation was found between VC levels and monocyte percentage. VE levels showed no significant correlations with any of the immune cell parameters (all P > 0.05).

**Table 4:** Correlation between vitamin levels, age, and inflammatory markers in pediatric patients with IBD.

Variables	WBC		Neutrophil (%)		Lymphocyte (%)		Monocyte (%)	
	r	P	r	P	r	P	r	P
VA (μmol/L)	-0.358	0.015	-0.500	<0.001	0.502	<0.001	0	0.997
VD (nmol/L)	-0.271	0.069	-0.374	0.011	0.396	0.007	0.007	0.962
VE (ug/ml)	-0.071	0.641	-0.101	0.505	0.115	0.447	0.098	0.518
VC (ug/ml)	-0.474	<0.001	-0.549	<0.001	0.512	<0.001	-0.248	0.097

IBD, inflammatory bowel disease; WBC, white blood cell count; VA, vitamin A; VD, vitamin D; VE, vitamin E; VC, vitamin C.

### Relationship between vitamin levels, nutritional status, and IBD

Univariate regression analysis identified SBP, heart rate, lymphocyte percentage, and monocyte percentage as factors associated with IBD. After adjusting for those factors, multivariate logistic regression was performed to assess the association between serum vitamin levels (and deficiency status) and IBD. For continuous vitamin levels, VA was significantly associated with IBD in both univariate and multivariate analyses (univariate: OR = 0.142, 95% CI: 0.046 - 0.440, P = 0.001; multivariate: OR = 0.159, 95% CI: 0.032 - 0.792, P = 0.025). No other continuous vitamin levels (VD, VE and VC) showed independent associations after multivariate adjustment (all P > 0.05). Regarding deficiency status, VA deficiency was significantly associated with IBD in both univariate (OR = 8.077, 95% CI: 3.177 - 20.537, P < 0.001) and multivariate (OR = 7.800, 95% CI: 1.735 - 35.055, P = 0.007) analyses. Deficiencies of VD, VE, and VC were not independently associated with IBD after multivariate adjustment (all P > 0.05) (**Table 5**).

**Table 5:** Association of vitamin levels and deficiency with IBD.

Variables	Univariate		Multivariate*	
	OR (95% CI)	P	OR (95% CI)	P
VA (μmol/L)	0.142 (0.046-0.440)	0.001	0.159 (0.032-0.792)	0.025
VA status				
VA sufficiency	1		1	
VA deficiency	8.077 (3.177-20.537)	<0.001	7.800 (1.735-35.055)	0.007
VD (nmol/L)	0.998 (0.978-1.018)	0.834	0.993 (0.959-1.029)	0.715
VD status				
VD sufficiency	1		1	
VD deficiency	2.533 (1.016-6.319)	<0.001	2.200 (0.528-9.162)	0.279
VE (ug/ml)	1.269 (1.019-1.579)	0.033	1.329 (0.949-1.861)	0.098
VE status				
VE sufficiency	1		1	
VE deficiency	1.726 (0.519-5.739)	0.373	1.621 (0.271-9.687)	0.596
VC (ug/ml)	0.934 (0.861-1.014)	0.104	0.948 (0.843-1.066)	0.373
VC status				
VC sufficiency	1		1	
VC deficiency	2.833 (1.180-6.803)	0.020	3.313 (0.753-14.576)	0.113

Adjusted for SBP, heart rate, lymphocyte percentage and monocyte percentage.

IBD, inflammatory bowel disease; SBP, systolic blood pressure; VA, vitamin A; VD, vitamin D; VE, vitamin E; VC, vitamin C.

## Discussion

The present study observes that pediatric patients with IBD exhibit significantly lower serum levels of VA and VC, along with higher VE levels, compared with healthy controls. Deficiency rates of VA, VC, and VD are also markedly increased in the IBD group. These findings align with previous reports. Notably, clinical studies specifically measuring circulating VA and VC concentrations in children with IBD remain scarce. Most available evidence is derived from adult cohorts, animal experiments or genetic epidemiological analyses, whereas high-quality pediatric clinical data are still limited (27–29). Prior studies have shown that VA deficiency (defined as serum VA < 20 μg/dL) occurs significantly more frequently in children with IBD compared with healthy controls, with an incidence ranging from 14% to 16%, and the risk is even higher in those with active CD (30, 31). In contrast, one pediatric IBD study investigating retinol-binding protein-4 (RBP-4, the major transport protein for VA) reported no significant inter-group differences between IBD children and healthy peers. This finding implies that alterations in VA transport, rather than circulating retinol concentration alone, should be considered when interpreting VA status in pediatric IBD (32). Our observed high VA deficiency rate (76.1%) further highlights the substantial burden of VA insufficiency among Chinese children with IBD, which cannot be fully extrapolated from existing Western pediatric data.

For vitamin C, adult IBD cohorts have reported 21.6% VC deficiency, especially among patients with elevated inflammatory markers or penetrating disease phenotypes (33). Direct pediatric clinical data for serum VC status in IBD are lacking. Adult data link higher inflammatory burden to worsening VC deficiency, and juvenile colitis animal models support VC-mediated anti-inflammatory and mucosal-repairing effects (29, 33, 34). Our 50.0% VC deficiency rate in children extends these adult observations to the pediatric setting and implies that children may suffer more profound VC depletion under intestinal inflammatory stress. Potential mechanisms underlying these deficiencies include reduced dietary intake, dietary restrictions, malabsorption, enhanced intestinal oxidative stress during active disease, and systemic inflammation (21, 35). A prior adult study reported inadequate dietary intake of VA, VC and VE among IBD patients (36). Moreover, elevated tumor necrosis factor-alpha (TNF-α) in IBD may downregulate the transcription of transporters required for VC absorption (37). Collectively, these findings underscore the clinical importance of systematic vitamin assessment in pediatric IBD. Leading clinical guidelines, including those from the European Society for Clinical Nutrition and Metabolism (ESPEN) and the European Society for Paediatric Gastroenterology, Hepatology and Nutrition (ESPGHAN), recommend routine nutritional monitoring in IBD patients, with regular evaluation of micronutrient status (38, 39).

From a nutritional-immunomodulatory perspective, within the IBD cohort, the present study also identified significant correlations between VA, VD and VC levels and circulating immune cell profiles (including white blood cell counts and differentials). As a well-established pleiotropic immunomodulator, 25-hydroxyvitamin D (25-OHD, the circulating biomarker of VD status) exerts broad anti-inflammatory, epithelial barrier-protective and gut microbiota-regulating effects in IBD pathogenesis via VDR signaling cascades, which form a core mechanistic basis for its bidirectional interaction with pediatric intestinal inflammation (40–42). Consistent with international pediatric cohorts, our study showed a high prevalence of VD insufficiency among Chinese children with IBD (43–45). Reported VD deficiency/insufficiency rates in pediatric IBD range from 52%-85% across studies, varying by cut-off values, treatment status and ethnicity (12, 43, 46). The 41.3% VD deficiency rate in our cohort is consistent with the high VD impairment frequently described in Asian pediatric IBD populations (47). Mechanistically, the active metabolite 1,25-dihydroxyvitamin D binds to VDR expressed on intestinal epithelial cells, macrophages, dendritic cells and T lymphocytes, suppressing pathogenic Th1 and T helper 17 cell (Th17) responses by inhibiting interferon-gamma (IFN-γ), interleukin-17 (IL-17) and interleukin-21 (IL-21), while facilitating Treg differentiation. It also restricts M1 macrophage polarization and promotes M2 phenotype, blunting interleukin-23 (IL-23) production-the central cytokine driving Th17-mediated intestinal inflammation in both pediatric CD and UC (42). Beyond immune tuning, VD strengthens intestinal barrier integrity by repressing chemokine (C-C motif) ligand 20 (CCL20) and upregulating antimicrobial peptides such as cathelicidin, which is elevated as a compensatory response in children with IBD (48, 49) and maintains gut microbial balance by supporting short-chain fatty acid-producing commensals (41).

VA, a diet-derived fat-soluble vitamin, exerts its immunoregulatory effects primarily through its active metabolite, RA. RA plays a critical role in maintaining mucosal immune homeostasis by synergizing with transforming growth factor-beta (TGF-β) to upregulate FOXP3, a master transcription factor for regulatory T cell (Treg) metabolism, thereby preserving the Th1/Th2 balance (16, 50). Inadequate RA availability skews naive CD4+ T cells toward a Th1 phenotype, promoting the release of pro-inflammatory IFN-γ and exacerbating intestinal immune imbalance and inflammation (51–53). VC (L-ascorbic acid), a water-soluble micronutrient absorbed primarily in the distal small intestine, exerts potent antioxidant effects, alleviating oxidative tissue injury and conferring protective effects against disease progression (54, 55). A recent study in a murine model of chronic colitis demonstrated that VC-loaded lipid nanoassemblies, which enhance targeted delivery and accumulation of VC at inflamed sites, attenuated immune cell-mediated inflammation and significantly ameliorated disease severity (56). Nevertheless, robust human pediatric evidence is still absent, emphasizing the need for further clinical investigation in children with IBD (29, 34). Collectively, VA, VD and VC appear to participate in the immune-inflammatory regulatory network of pediatric IBD, although precise *in-vivo* mechanisms remain to be fully elucidated.

A growing body of evidence indicates that VD status in pediatric IBD is closely tied to extraintestinal complications and genetic susceptibility, rather than serving as a direct marker of mucosal inflammation. Cakar S et al. (12) found that 85% of UC and 81% of CD patients had sarcopenia, and those with more severe muscle wasting had significantly lower VD levels (median 9.8 vs. 16.9 ng/mL) in newly diagnosed children, reinforcing earlier data that severe VD insufficiency is independently correlated with reduced muscle mass at diagnosis. One pediatric study has documented a dissociation between circulating 25-OHD and endoscopic or clinical disease-activity indices, supporting a predominantly systemic, extra-intestinal protective role for VD in children (57). However, cross-sectional and prospective pediatric studies consistently fail to detect linear correlations between circulating 25-OHD and fecal calprotectin, endoscopic severity, or clinical disease activity indices (58, 59), suggesting that VD exerts systemic protective effects (e.g., ameliorating iron deficiency, mitigating muscle wasting) rather than directly suppressing intestinal inflammation in children. Furthermore, VDR gene polymorphisms may modulate individual risk: the caudal type homeobox 2 (Cdx2) and BsmI heterozygotes were associated with significantly higher pediatric IBD risk, and the FokI polymorphism was linked to disease activity in UC (11). This may partly explain the present finding that VD deficiency rates, but not absolute VD levels, differed between groups, as local healthy children already exhibit a high background VD deficiency rate of 41% (24), attenuating the between-group difference in levels and highlighting that VD status should be interpreted in clinical context rather than as a stand-alone disease biomarker. Epidemiological data consistently report extreme VD deficiency prevalence ranging from 42.5% to 91.5% across multi-regional IBD cohorts, with female sex and younger age at diagnosis identified as independent risk factors for profound 25-OHD insufficiency-features highly relevant to pediatric populations (60, 61). In real-world practice, a multicenter survey study found that 81.4% of parents of children with IBD administered dietary supplements, with VD being the most frequently supplemented, predominantly based on physician recommendation (62). Observational studies further validate that VD supplementation reduces IBD-related emergency visits, hospitalizations and cumulative corticosteroid exposure, supporting its role as a low-cost adjuvant therapy, even though high-quality pediatric randomized controlled trials are still needed to confirm its efficacy in inducing mucosal remission (63, 64).

Multivariable regression analyses further suggest potential independent associations between low serum VA levels, VA deficiency and pediatric IBD. Notably, these findings appear inconsistent with a previous investigation of 87 IBD patients, which linked higher dietary VA intake to increased IBD prevalence (65). Genetic-epidemiological studies have yielded contradictory findings: Mendelian-randomization work indicated higher circulating retinol may lower UC risk, whereas case-control studies reported higher IBD risk with greater dietary retinol intake (27, 28). These discrepancies may originate from differences between questionnaire-based dietary intake versus directly measured serum concentrations, ethnic and age heterogeneity, as well as the dual pro- and anti-inflammatory properties of RA metabolites (66, 67). Beyond measurement differences, VA biology is context-dependent: under inflammation, RA-primed dendritic cells can promote pro-inflammatory Th1/Th17 polarization (68). Therefore, in IBD, VA deficiency may compromise mucosal barrier function and immune regulation, facilitating disease onset; conversely, elevated systemic VA levels may exert proinflammatory effects and thus also confer potential disease risks (16). Reif S et al. reported that a higher intake of retinol was positively associated with the risk for IBD (65). Collectively, these observations indicate that VA supplementation in pediatric IBD patients should be implemented cautiously, favoring individualized precision strategies with close monitoring of serum VA concentrations to avoid potential adverse effects associated with excessive VA exposure.

Notably, the present study extended vitamin assessment to include B-group vitamins (B1, B2, B3, B5), which primarily function as coenzymes in cellular energy metabolism and other fundamental biological processes (69). No statistically significant differences in levels or deficiency rates of B vitamins were observed between pediatric IBD patients and healthy controls, suggesting that B vitamin deficiency is not a universal feature of IBD in this cohort. Additionally, no significant differences in vitamin levels or deficiency rates are observed between CD and UC. However, this finding requires validation in larger, adequately powered nutritional studies specifically designed for each disease type.

In summary, the present study observes that pediatric IBD patients have significantly lower serum VA and VC levels and higher VE levels compared with healthy controls, with markedly elevated deficiency rates of VA (76.1%), VC (50.0%), and VD (41.3%). Since direct pediatric comparative data for VA and VC remain sparse, our work adds real-world observational evidence describing multi-vitamin nutritional profiles among Chinese children with IBD. Within the IBD cohort, VA, VD, and VC levels were all significantly negatively correlated with neutrophil percentage and positively correlated with lymphocyte percentage. Multivariate logistic regression further identified low serum VA level and VA deficiency as independent factors associated with IBD. These results provide a comprehensive characterization of the multi-vitamin profile in pediatric IBD and highlight the close interplay between specific vitamin deficiencies and systemic immune cell alterations.

Nevertheless, several limitations warrant consideration when interpreting the findings. First, this single-center retrospective study had a modest sample size, potentially introducing selection bias and limiting generalizability. Because vitamin profiling was recommended according to guidelines/consensus and performed with guardian consent rather than as universal screening, only children who underwent multiplex vitamin profiling were consecutively enrolled, whereas those without multiplex vitamin data were excluded. Selection bias therefore cannot be excluded, deficiency prevalence may be overestimated or underestimated, and included and non-included patients could not be formally compared. The modest sample size also resulted in wide 95% confidence intervals and insufficient statistical power for some analyses, suggesting possible overestimation of effect sizes that requires validation in larger cohorts. Second, CD and UC are distinct disease entities rather than disease subtypes. Our sample size for separate analyses comparing between CD and UC is limited, leading to insufficient statistical power for direct head-to-head comparisons. Accordingly, larger, adequately-powered multicenter studies are required to independently evaluate vitamin nutritional status in CD and UC, respectively. Third, potential confounders, including detailed dietary intake and dietary patterns, were not fully accounted for, which could have introduced bias. Fourth, VDR gene polymorphisms and other genetic factors were not genotyped, precluding analysis of gene-vitamin interactions. Fifth, nationally-unified reference standards for defining insufficiency of several vitamins are lacking for Chinese children. We adopted overseas clinical laboratory thresholds in the present study, which may lead to over- or under-estimation of vitamin deficiency rates. Of note, comparisons of continuous serum vitamin concentrations are independent of these categorical deficiency cut-offs. Finally, the cross-sectional design precludes any causal inference regarding the relationship between VA levels and IBD risk; prospective cohort studies are needed to address this question.

## Conclusion

In conclusion, this study observes lower serum VA and VC levels in children with IBD relative to healthy controls. Within the IBD cohort, VA, VC and VD are significantly correlated with peripheral immune-cell proportions. Multivariate regression suggests potential independent associations between low VA status and IBD. Given the single-center retrospective design and limited sample size, these are preliminary observations requiring confirmation in larger multicenter studies. Our findings support comprehensive vitamin evaluation as part of pediatric IBD clinical management, particularly addressing the prevalent deficiencies of VA, VC, and VD.

## Data Availability

The original contributions presented in the study are included in the article/supplementary material. Further inquiries can be directed to the corresponding authors.
